# Goal-Concordant Care in the Era of Advanced Stroke Therapies

**DOI:** 10.1089/jpm.2019.0667

**Published:** 2021-01-15

**Authors:** Leonard L. Sokol, Joshua M. Hauser, Hillary D. Lum, Jodi Forlizzi, Moran Cerf, Fan Z. Caprio, Michael J. Young

**Affiliations:** ^1^ The Ken and Ruth Davee Department of Neurology, The Ken and Ruth Davee Department of Neurology, Northwestern University Feinberg School of Medicine, Chicago, Illinois, USA.; ^2^ McGaw Bioethics Scholars Program, Center for Bioethics and Humanities, The Ken and Ruth Davee Department of Neurology, Northwestern University Feinberg School of Medicine, Chicago, Illinois, USA.; ^3^ Center for Bioethics and Medical Humanities, Institute for Public Health and Medicine, The Ken and Ruth Davee Department of Neurology, Northwestern University Feinberg School of Medicine, Chicago, Illinois, USA.; ^4^ Section of Palliative Medicine, Department of Medicine, The Ken and Ruth Davee Department of Neurology, Northwestern University Feinberg School of Medicine, Chicago, Illinois, USA.; ^5^ Palliative Care Service, Jesse Brown VA Medical Center, Chicago, Illinois, USA.; ^6^ Division of Geriatric Medicine, University of Colorado School of Medicine, Aurora, Colorado, USA.; ^7^ Eastern Colorado VA Geriatric Research Education and Clinical Center, Rocky Mountain Regional VA Medical Center, Aurora, Colorado, USA.; ^8^ Human-Computer Interaction Institute, School of Computer Science, Carnegie Mellon University, Pittsburgh, Pennsylvania, USA.; ^9^ Interdepartmental Neuroscience Program, Northwestern University, Evanston, Illinois, USA.; ^10^ Kellogg School of Management, Northwestern University, Evanston, Illinois, USA.; ^11^ Division of Stroke and Neurocritical Care, The Ken and Ruth Davee Department of Neurology, Northwestern University Feinberg School of Medicine, Chicago, Illinois, USA.; ^12^ Department of Neurology, Harvard Medical School, Massachusetts General Hospital, Boston, Massachusetts, USA.

**Keywords:** advance care planning, goals of care, hospital transfers, stroke, thrombectomy, withdrawal of interventions

## Abstract

Stroke is a leading cause of disability and mortality worldwide. Recent advances in stroke care now enable patients with severe ischemic stroke owing to large vessel occlusion to safely undergo endovascular thrombectomy (EVT) up to 24 hours since their time of last known well, with the goal of improving functional outcomes by recanalization of the occluded vessel and reperfusion of downstream ischemic brain tissue. The objective of this analysis is to highlight clinical and ethical challenges related to ensuring goal-concordant care in this era of unprecedented advances in acute stroke care. Specifically, there is a salient challenge of whether advanced therapies such as EVT may be justifiably considered comfort focused, given their potential to preempt accumulated neurologic disability and suffering at the end of life. Through the lens of a patient case, we discuss key challenges, lessons learned, and suggestions for future care and research endeavors at the intersection of acute stroke care and palliative care principles. Although therapies such as thrombolysis and EVT may be considered aggressive *prima facie*, their potential to ameliorate additional disability and potential suffering at the end of life prompt close consideration of the proper role of these therapies on a case-by-case basis in the context of comfort-focused care. Modification to the workflow for EVT evaluations may facilitate goal-concordant care and timely resource allocation, especially for cases that involve hospital-to-hospital transfers for advanced stroke care.

## Introduction

Stroke is a leading source of disability and mortality in the world, second only to heart disease.^1^ Although intravenous thrombolysis with tissue plasminogen activator (tPA) is the mainstay of acute ischemic stroke therapy, only approximately 5% of patients are eligible for such treatment.^1^ Time limitations and other narrow inclusion criteria may contribute to low eligibility, as under present guidelines clinicians can only administer tPA if patients present within 4.5 hours since their last known well time.^2^ Unlike tPA, which delivers a systemic blood-thinning medication to alleviate cerebral clot burden, the goal of endovascular thrombectomy (EVT) is the mechanical removal of a clot to recanalize the occluded vessel and reperfuse downstream ischemic brain tissue. The use of EVT has revolutionized the acute treatment of stroke, as such intervention can occur within 24 hours after the patient's last known well time.^3–5^

Novel clinical and ethical challenges arise in the context of ensuring goal-concordant care as acute stroke care advances, including the challenge of whether advanced therapies such as EVT may be justifiably considered comfort focused given the potential to prevent significant neurological disability and suffering at the end of life. Although therapies such as thrombolysis and EVT may be considered aggressive *prima facie,* their potential to reduce poststroke suffering at the end of life warrants careful consideration of the potential palliative benefit as part of comfort-focused care. To illustrate these challenges, we discuss the case of a patient with a large vessel occlusion of the right middle cerebral artery (MCA), transferred for an EVT evaluation, for whom the medical team learned only after the transfer that the patient's preferences were for comfort-focused treatments. We consider the value of exploring goals of care before hospital transfers and further define the normative valence of novel acute stroke therapies such as EVT within operationalized treatment categories (i.e., full treatment, selective treatment, and comfort-focused treatment).

## Case Description

A previously healthy 85-year-old left-handed man developed sudden-onset left facial droop, left arm weakness, global aphasia, and rightward gaze deviation. Minutes after the onset of symptoms, emergency services were activated, and transferred the patient to the nearest hospital, where he was diagnosed with an ischemic stroke localizable to the right MCA territory. As the patient was within the 4.5-hour window of his last known well and had no contraindications, he immediately received tPA.

He was transferred to a tertiary care center for consideration of EVT. The patient's examination upon arrival to the tertiary center was unchanged with a National Institutes of Health Stroke Scale (NIHSS) of 20, indicating a severe right MCA syndrome. CT angiography demonstrated a thrombus in the proximal, right MCA; perfusion imaging indicated a sizeable ischemic penumbra (i.e., a vast territory of brain tissue at risk of further irreversible damage if blood flow is not achieved).

The patient was widowed, had one daughter, and no designated health care power of attorney or other advance directives. The medical team reached the daughter, his default surrogate decision maker (surrogate), through phone. This process explored the daughter's understanding of the situation and informed her of the possibility of surgical intervention. The daughter understood that her father had had a stroke and was transported to a different hospital.

The series of diagnostic and therapeutic measures were briefly reviewed, including the use of tPA. She was informed that the stroke had affected a large vessel within the brain that governed functions such as attention, articulation, swallowing, and left-sided sensation and movement. The daughter indicated that at no point had she consented to tPA on her father's behalf (in emergency care settings, a model of presumed consent for emergency therapies, such as tPA, is common),^6,7^ and was unclear why her father was transferred. The medical team underscored that, although tPA was one type of acute stroke therapy, EVT could also potentially remove the clot, possibly decreasing his neurologic deficits and preventing further disability.

His daughter highlighted that several months before his admission he had expressed a preference for a do-not-resuscitate (DNR) order when admitted to the hospital for viral gastroenteritis. It was clarified that DNR is not equivalent to “do not treat” or even synonymous with only comfort-focused treatment,^8^ and that one could still have a DNR order and receive medical interventions to prolong or sustain life. Nevertheless, his daughter unequivocally indicated that her father should not receive any medical interventions aimed at prolonging life, in line with her understanding of his prior wishes. She predicated this on his recent behaviors and conversations, including his refusal to take anticoagulation despite a cardiologist's recommendations, declining a pacemaker a year before his stroke, and consistent opposition to medical procedures. In line with this understanding of the patient's preferences and goals, he was treated for symptoms of discomfort and transitioned home with hospice services. The palliative medicine service was consulted, and also ensured that the surrogate participated in informed shared decision making with proper understanding, appreciation, and communication around the reasoning for forgoing life-sustaining treatments.^8^

## Discussion

EVT evaluations are unique, as they often involve a partnership between academic and community clinicians, through virtual interactions like telestroke, and sometimes involve hospital transfers. Because potentially appropriate patients often have very disabling deficits, as few as 17% of patients may be able to provide consent.^9^ In patients eligible for EVT, there are limited data estimating the proportion of cases where patients or surrogates forgo the intervention and why.

In this case, because the patient exhibited nearly all the clinical attributes requisite for an EVT, the medical team initially responded skeptically to the surrogate's judgment that her father would not want this intervention. This sentiment likely arose from two factors. First, between 34% and 44% of patients who undergo EVT experience disability reduction, whereas only 1% suffer a worsening of disability.^10^ Second, the refusal of EVT appeared contrary to the preceding therapeutic momentum. The patient had first been rushed to the community hospital, received tPA, and then finally transferred to an academic center for EVT. Only at that point was the surrogate asked about goals of care, and compellingly voiced that the patient preferred comfort-focused treatments and would be opposed to any procedure. Team members worked to ensure that the surrogate's articulated choice was consistent with the patient's long-standing values, preferences, and goals.

This case illustrates four salient challenges. First, no formalized advance care planning documentation or portal medical orders, such as a Physician's Orders for Life-Sustaining Treatment (POLST) form, were available, which may have facilitated goal-concordant care from the onset of symptoms. The challenges associated with ensuring compassionate, goal-concordant care for patients who are incapacitated are many and amplified in settings where conflicts might exist between clinician perspectives and the substituted judgment of the surrogate.^11^ Second, the case was medically appropriate for EVT, which may have generated erroneous assumptions about the patient's preferences and impeded optimal communication. Third, exploration of the patient's goals of care as part of clinical decision making for advanced stroke treatment and consideration of EVT occurred after transfer. Because the patient's preferences were unknown before the transfer, there was delayed achievement of goal-concordant care and corresponding inappropriate allocation of resources. Fourth, the choice of comfort-focused treatment did not necessarily preclude the use of EVT.^12^ This case highlights the lack of clarity and consensus about whether EVT, as an invasive procedure, can be justifiably conceptualized as promoting comfort.

In discussing the fourth challenge, it is helpful to reference the National Institute on Aging's definition of comfort care.^12^ It defines it as “…care that helps or soothes a person who is dying… to prevent or relieve suffering as much as possible and to improve quality of life while respecting the dying person's wishes.”^13^ Scholars have commented on the ambiguity of the phrase, as invasive measures may still help to achieve these aims.^12^ Indeed, EVT is a fitting example. Successful EVT often results in the reduction of aphasia or pain, the return of strength or cognition, and thus may improve quality of life, analogous to the use of palliative radiation. Although the surrogate argued against all interventions for the patient, other patients with a similar clinical presentation and preferences for comfort-focused treatment could foreseeably and justifiably agree to EVT.

Consider, for example, a cancer patient with an estimated 1-year prognosis who has decided to focus on comfort. However, shortly after that decision, the patient suffers a right MCA stroke syndrome. In the appropriate context, the clinical role of EVT could become a question of whether that person would want to live the remaining time paralyzed and aphasic, or with minimal deficits.

Taken together, the four challenges may be surmounted with a modification to the workflow for EVT evaluation. Aligned with other proposals regarding informed consent for EVT, we also suggest that any member from the medical team, including a nurse, social worker, or chaplain, should discuss the potential of advanced stroke therapies with the surrogate as early as the patient arrives with manifestations consistent with a large vessel occlusion.^14^ Miscommunication contributed to 60% of inappropriate hospital transfers, and data suggest that interaction with the surrogate impacts medical decision making.^15^ Thus, before a transfer to another hospital for EVT, timely communication with the surrogate ought to be a priority.

Time is of the essence in large vessel occlusion strokes, wherein it is estimated that approximately two million neurons are lost for every minute delay in treatment.^10^ Clinicians must, therefore, balance high-quality consent without harming the patient or surrogate. One component that may be helpful is a communication framework for emergency surgical interventions.^16^ Dialogue conveyed from the medical team would include themes such as prognosis, information, options, and goals.^16^ A team member may assess the surrogate's understanding of the situation (information) and then inquire about the surrogate's substituted judgment (goals). The inquiry might be phrased as follows: “What would the individual *think/say* about having an intervention to remove the clot which might restore function?” Past data demonstrate benefits in using the words “think” or “say” over “want” in this context.^17^

Another component that may enhance the informed consent process is the use of a decision aid.^10^ Decision aids, also commonly used during consent for thrombolytic therapy for acute ischemic stroke, may be delivered electronically or in-person. The optimal visual aid would capture the immediate complications (e.g., dissection and hemorrhagic conversion) and high-level, long-term benefits/risks while avoiding jargon.^10^ Such a tool, which adheres to the guidelines and best practices for consent during emergency scenarios, has already been engineered.^10^ This two-pronged approach—a communication framework and a decision aid—may help to ensure that the patient's values are optimally captured and honored, and further prospective studies to evaluate goal concordance in the acute stroke setting can strengthen paradigms of care.^18^

In the optimal scenario, advance care planning documentation would have been available and easily accessible. Ideally, the medical team would have been able to contact the surrogate, review the patient's stated preferences, and clarify whether EVT was aligned with promoting the patient's goals. Such measures may also obviate the misallocation of resources, which in the forgoing case was only realized in retrospect. Even if advance care planning documentation were available, there still would be appropriate tolerance for a hospital transfer from home to ensure comfort. Indeed, POLST forms permit such malleability as aligned with comfort-focused care.^15^

A reconsideration of the workflow can help safeguard the appropriateness of acute stroke transfers.^19^ Because only limited tertiary centers perform EVTs, further longitudinal studies are needed to explore how to ascertain a patient's values rapidly and ensure goal-concordant care. Decision support tools can use or infer patient preferences based on historical information, classification to similar patients (in the absence of information), or impute data about patients based on extrapolation of existing information.^20–22^ These are likely to yield outcomes that are more aligned with expected patient preferences.^23^

However, these systems cannot take into account the essential human judgments that surround care, primarily from the clinician's perspective.^24^ They raise ethical questions about the tradeoffs between individual, institutional, and societal benefits and the costs of using technology to improve human judgments. That is, whether the scenario is one where the heuristics fall short of the individual's performance, and that is the reason that the individual prefers not to use its recommendation. Alternatively, inherent biases exist against technological solutions—irrespective of the actual performance—that lead stakeholders to refrain from using them.

Future work also ought to explore the number of hospital-to-hospital transfers that involve an escalation of care, those that independent evaluators might judge as at odds with patient preferences, and processes to prevent potentially unwanted transfers. Although therapies such as thrombolysis and EVT may be considered aggressive prima facie, their potential to improve quality of life and ameliorate further suffering at the end of life warrants closer consideration of the proper role of these therapies in the context of comfort-focused care on a case-by-case basis.
